# UAV Trajectory Optimization in a Post-Disaster Area Using Dual Energy-Aware Bandits †

**DOI:** 10.3390/s23031402

**Published:** 2023-01-26

**Authors:** Amr Amrallah, Ehab Mahmoud Mohamed, Gia Khanh Tran, Kei Sakaguchi

**Affiliations:** 1 Department of Electrical and Electronic Engineering, School of Engineering, Tokyo Institute of Technology, 2-12-1 Ookayama, Meguro-ku, Tokyo 152-8550, Japan; 2 Academy for Super Smart Society, Tokyo Institute of Technology, 2-12-1 Ookayama, Meguro-ku, Tokyo 152-8550, Japan; 3 Department of Electrical Engineering, College of Engineering in Wadi Addawasir, Prince Sattam Bin Abdulaziz University, Wadi Addawasir 11991, Saudi Arabia; 4 Department of Electrical Engineering, Faculty of Engineering, Aswan University, Aswan 81542, Egypt

**Keywords:** unmanned aerial vehicle, trajectory optimization, reinforcement learning, multi-armed bandit, cost subsidy, post-disaster

## Abstract

Over the past few years, with the rapid increase in the number of natural disasters, the need to provide smart emergency wireless communication services has become crucial. Unmanned aerial Vehicles (UAVs) have gained much attention as promising candidates due to their unprecedented capabilities and broad flexibility. In this paper, we investigate a UAV-based emergency wireless communication network for a post-disaster area. Our optimization problem aims to optimize the UAV’s flight trajectory to maximize the number of visited ground users during the flight period. Then, a dual cost-aware multi-armed bandit algorithm is adopted to tackle this problem under the limited available energy for both the UAV and ground users. Simulation results show that the proposed algorithm could solve the optimization problem and maximize the achievable throughput under these energy constraints.

## 1. Introduction

Across the globe, large-scale natural disasters are known for their severe casualties damage to property. Besides thousands of deaths and injuries resulting from various types of natural disasters around the world, there has been additional increase in material losses of about 100–150% [1]. The first few hours after a catastrophe are regarded as the “golden hours” of relief because rescue workers have a high probability of evacuating people from the damaged region during this period. Keep in mind that the wireless infrastructure in the disaster area might not be functional or even might be ravaged after the disaster. What makes the situation even more complicated is the paralysis of the power transmission lines after the disaster. The most powerful earthquake ever recorded in Japan, with a magnitude of 9.1, triggered a tsunami on the northeastern shore in March 2011. In the region of the catastrophe, around 6000 base stations (BSs) were wrecked, and the remaining BSs were highly overloaded with tremendous amounts of voice and data traffic. As a result of the high call block rate, communication services were suspended for roughly four days [2]. As a result, it is critical to develop an emergency wireless network that is completely independent of the conventional broadband network as soon as possible in order to preserve those valuable human lives. Unmanned aerial vehicles (UAVs) are well-known for their distinct characteristics, such as flexible deployment and rapid reaction. Thus, they can be deployed as temporary mobile BSs to establish this type of temporary emergency wireless network [3]. UAVs are now employed for a variety of emergency wireless communication applications, such as disaster management, surveillance, early warnings, post-disaster fusion centers, damage assessment, and supply-aid drop, in addition to temporary emergency wireless networks [4].

Notwithstanding the advantages of utilizing UAVs for establishing emergency wireless communication networks in a post-disaster area, there are a number of issues that need to be neutralized. In this tough environment induced by a natural disaster, the UAV must first design and optimize its flying route. This necessitates a quick online optimization procedure to accommodate the dramatic shift in the geographical field [5]. Secondly, the available energy for victims is ephemeral due to the limited battery capacity of their UEs and the destruction of the power supply infrastructure as a result of the natural disaster [6]. Thirdly, the UAV’s operating duration is restricted by the onboard battery’s capacity. The UAV should return to its base for recharging before it is completely depleted [7]. Therefore, while constructing an emergency wireless communication network, all of these concerns should be addressed. In addition, since this is a crucial mission, the UAV must assist as many people as possible in the disaster zone before its battery dies. Consequently, it is vital to seek out a robust mathematical tool capable of tackling such novel challenges.

Machine learning (ML) algorithms, and more precisely, reinforcement learning (RL) algorithms, are leveraged to tackle these kinds of optimization problems. Since RL algorithms are capable of achieving superb results in terms of efficiency and generalization, and due to their ability to deal with optimization problems with conflicting parameters, researchers have been inspired to utilize them in dealing with real-time issues in the field of wireless communications networks [8]. In this context, modern UAVs are equipped with wireless communications, ML, and image processing techniques. These techniques can support a UAV’s trajectory optimization while avoiding obstacles and dealing with a limited battery capacity, which leads to serving more spots and enhancing the whole mission’s energy efficiency. Recently, “follow me” drones have boomed in market value [9]. These drones are capable of filming a moving person with intelligent target-tracking and obstacle-avoidance algorithms, resulting in fabulous camera footage. Furthermore, novel UAV-related applications such as area surveillance, disaster relief, and traffic control are just a few applications that can be intelligently developed for future cities [10].

Multi-armed bandit (MAB) algorithms are considered one of the RL algorithms which are preferred in dealing with online optimization problems [11]. MAB algorithms can be defined as a set of arms, i.e., actions, of a bandit machine. At any given moment, pulling an arm leads to an instantaneous reward that is sampled from a certain distribution. A player wants to maximize his accumulated reward over the playing period by choosing an arm to pull during each moment of playing. Nevertheless, this player has no idea about the instantaneous reward behind each arm, since it will be revealed when the player decides to choose it. Therefore, some amount of the reward could be missed out due to this hidden setting. This loss is denoted by the term regret [12,13]. Thus, a player should develop a strategy to choose the arm that leads to the highest reward. On the other hand, this strategy should keep an eye on balancing between playing with the previously discovered arms that have high rewards or playing with the still-undiscovered ones that might have higher rewards. This is a common MAB dilemma, and it is called the exploration–exploitation trade-off [14,15]. Aiming to bolster disaster resilience, this paper describes a method of leveraging the latest advances in MAB algorithms and UAV wireless communications networks to improve the functionality of emergency wireless communication services for post-disaster response and assessment.

### 1.1. Prior Works and Motivations

One of the main benefits of deploying UAVs in emergency wireless communication networks is their capability of gathering extensive data from scattered ground devices, such as ground BSs, ground users, and even ground sensors [16]. The paper just cited gives a broad overview of different techniques but does not dive deeply in a specific direction. Furthermore, a UAV can operate as a flying edge server or a BS to support various traffic offloading scenarios [17], but it has a limited size of state action space. Due to its mobility, the planning and optimization of the UAV’s trajectory and radio resource management of its wireless network are crucial issues. Researchers conducted many investigations on this topic during the past few years [18]. The UAV’s speed and the location of its waypoints were used in [19] to design an optimal trajectory. However, the discussion was limited to cases where UAVs are used as relay stations in ad hoc networks. Minimizing the total energy consumption was studied in [20] using UAV speed control and a UAV data-scheduling-based heuristic algorithm, but it can be considered a theoretical approach only due to its large approximation factors. The authors of [21] considered UAVs with small cell capabilities to work as UAV-BSs. Particularly, the UAV movement, charging, and coverage action are considered in terms of jointly optimizing the energy and throughput through revenue and cost components. The UAV task scheduling was investigated in [22], where a mathematical framework for the optimization of UAV-aided video monitoring of a set of points of interest (PoI) distributed in a large urban area was proposed. Using this framework, which is based on mixed integer linear programming (MILP) techniques and real experimental data, particular energy-constrained UAVs are selected for recharging using public transportation buses, which also transfer the UAVs to desired PoIs in order to increase reliability and coverage.

UAV trajectory optimization may be carried out using traditional optimization approaches when realistic models of UAV wireless networks, including their flight dynamics, are available. Still, building these realistic network models is quite challenging; thus, model-free machine-learning methods can be used to manage the operation of UAVs that utilize wireless communication networks. By utilizing data gathered from prior experiences, machine learning algorithms are able to create autonomous control policies [23]. The authors of [24] studied the optimal deployment of UAVs equipped with directional antennas, using circle packing theory, where the 3D locations of the UAVs are determined such in a way that the total coverage area is maximized. The policy gradient approach for trajectory optimization used by the authors of [25] was able to maximize the overall distance covered by the UAV. However, this method took a lot of time and effort to find the best answer due to the large number of possible trajectories that the UAV must fly. The authors of [26] used the deep Q-learning method to optimize the UAV’s flight path to maximize data rate during the flight period in an unknown environment. One major limitation of this proposed Q-learning approach for trajectory optimization is the long learning time, which makes it unfeasible even for moderate state spaces. By planning the UAV’s flight trajectory, the authors of [27] were able to maximize the uplink transmission rate in a UAV cellular network. The deterministic policy gradient (DPG) approach was used to solve the optimization problem after it was converted into a Markov decision process (MDP). However, the characteristics of mmWave channels and beamforming were not taken into consideration during the optimization process.

Despite the existence of numerous excellent studies on UAV wireless communication networks, there are only a few works that focus on UAV-assisted emergency wireless communication networks. In our earlier studies [28,29], we investigated the radio resource allocation for a UAV emergency wireless communication network using a dynamic spectrum access system. The purpose of the deployment of UAVs as a cognitive radio network (CRN) was to maximize the downlink data rate in a post-disaster environment. Moreover, the limited transmission power of each UAV was used to control the constructed two multi-player MAB-based optimization problems called the power budget aware upper confidence bound (PBA-UCB) algorithm and the power budget aware Thompson sampling (PBA-TS) algorithm. The problem of gateway selection in a post-disaster area was addressed in [30], where a decentralized MAB algorithm was adapted to each UAV to let it maximize its data throughput by optimally choosing a suitable gateway. However, the optimization algorithm encountered some data loss due to not choosing the optimal strategy at the beginning of the optimization process. The authors of [31] built a system of a re-configurable intelligent surface (RIS) attached to a UAV. With the aid of a modified version of the MAB algorithm, the optimization problem aimed to find the optimum trajectory of the UAV that maximizes the total throughput while reducing the consumed flying power of the UAV. For a UAV with a limited battery capacity, the maximization problem for the number of served users was studied in [32] using two MAB algorithms called the ϵ-greedy algorithm and the D-UCB algorithm. The UAV trajectory optimization problem was studied in [33] to maximize the accumulated data volume from ground sensors under unknown network information. The optimization problem was transformed into a finite MDP and solved using two Q-learning-based UAV trajectory optimization frameworks called SUTOA and QUTOA. A Lyapunov-based deep Q-learning framed work called Safe-DQN was proposed in [34] to study the UAV trajectory optimization problem in a UAV-based emergency wireless communication network. The joint optimization problem aimed to maximize the total system rate under the constraints of the limited flight time of the UAV, the power capacity of the ground user, and the need to avoid obstacles in the disaster area. All the previous research was controlled by the limited capacity of the attached onboard battery for each UAV.

All of these studies on UAV emergency wireless communication networks focused on the optimization issue under a single power restriction, either a restricted UAV battery capacity or a limited amount of energy accessible to ground users (i.e., ground UE or ground sensors). We argue that these two elements together should be taken into account while constructing a UAV emergency wireless communication network. This is because the natural disaster destroys or at least renders the power supply network inoperable. Therefore, the goal of our suggested framework is to solve the UAV trajectory optimization problem under these two limited power constraints. In order to do this, our goal was to investigate a dual constraint optimization problem that might increase the UAV emergency wireless network’s reliability in comparison to earlier studies. It should be noted that, to the best of our knowledge, our earlier work in [35] was the first study to investigate this sort of optimization issue with dual constrained energy capacity for both UAV and UEs at the same time. Furthermore, in the research, we extend our problem formulation by deeply evaluating the performance of our proposed framework against different benchmark methods. This evaluation was conducted in terms of the accumulated long-term uplink throughput of all UEs, the energy consumed by all UEs during the data-offloading process, and the energy efficiency of the UEs.

### 1.2. Contributions and Organization

According to the discussion in the preceding subsection, the majority of recent research on UAV emergency wireless communication networks concentrated primarily on the limited battery energy capacity of UAVs; just a small number of studies took into account the restricted energy capacity of ground users, i.e., ground users’ equipment (UEs). We created a suggestion to fill this gap by examining an optimization scenario with constrained energy capacity for both UEs and UAVs. UAVs are seen as flying BSs that provide a wireless connection to ground UEs in the disaster-affected region from the sky. The information gathered from the UEs is deemed critical for estimating the status of the victims and assessing the damage in the post-disaster area. As a result, this critical data may be processed to help rescue crews save these precious lives. Our major goal is to acquire as much data from ground UEs as possible given the restricted power capacity of both the UAV and the ground UEs. However, since UAV coverage is somewhat limited in comparison to terrestrial BSs, our goal is to optimize the UAV flight trajectory to maximize the number of ground UEs visited before the battery runs out. Considering this limited battery capacity, another interesting idea is to have the UAVs maximize the scanned area while capturing photos to aid the rescue teams or to estimate the damage caused by the natural disaster. This goal was kept for our future work. The primary contributions of this work can be summarized as follows:

- In our situation, a UAV would gather user data in a disaster-affected region as part of a wireless emergency communication network. Ground BSs fail as a consequence of natural catastrophe damage, but ground UEs in the UAV coverage area may upload data using an alternate mode of connection from the sky thanks to the assistance of the UAV emergency wireless communication network. We propose an online optimization problem to optimize the uplink throughput for the UAV emergency wireless communication network by optimizing the flight trajectory of the UAV under these assumptions, taking into consideration the limited available energy for both the UAV and ground UEs in the post-disaster region.
- The optimization problem is adapted into a constrained MAB problem, with action, reward, and cost defined as the flight direction, uploaded data throughput, and dissipated energy for both the UAV and UEs, respectively.
- The numerical analysis of our proposed framework shows a considerable increase in long-term throughput and a slight increase in the energy consumption of the UEs in the post-disaster area, resulting in better energy efficiency for our proposed framework compared to other benchmark UAV trajectory optimization methods.

The rest of this paper is organized as follows. Section 2 presents the network architecture and formulates the online optimization problem for the long-term uplink throughput maximization problem. In Section 3, the general MAB framework is illustrated, followed by our proposed MAB-based framework for UAV trajectory optimization under dual energy constraints. Simulation results and numerical analysis are given in Section 4, and finally, the paper is concluded in Section 5.

## 2. Network Architecture and Problem Formulation

In this section, we discuss the architecture for the UAV-assisted emergency wireless communication network, including the flying model used for the UAV, the channel model for data uploading, and the optimization problem formulation.

### 2.1. UAV Flying Model

**Figure 1:**
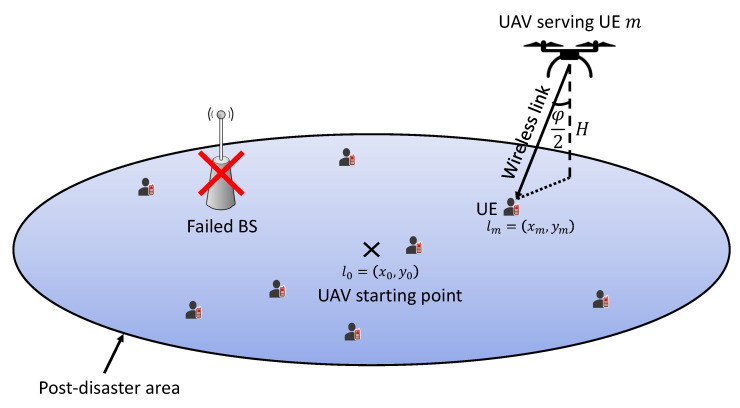
UAV emergency wireless communication network.

The system architecture for the UAV-assisted emergency wireless communication network is shown in Figure 1. In this scenario, a natural disaster, such as an earthquake or flood, strikes a specific location and causes the power grid and wireless network to fail. Our plan is to use the UAV to enable wireless access from the sky in this post-disaster area. In this approach, wireless connectivity may be enabled for victims, i.e., ground UEs, in this devastated region, allowing them to offload data that will be useful in guiding rescue crews and evaluating the damage. We assumed that there are *M* UEs trapped in this post-disaster area, denoted by M={1,…,M}. Each of them has a fixed position designated by the following in Cartesian coordinates $l_{m}=\left(x_{m},y_{m}\right)$. The UEs locations are supposed to be known to the UAV through self-reported global positioning system (GPS) coordinates. The discussion on how these data are transferred to the UAV is beyond the scope of this paper. It is assumed that the UAV will begin flying from the center of the post-disaster area, i.e., the center of the simulation area, which is denoted by $l_{0}=\left(x_{0},y_{0}\right)$. Additionally, it flies according to a constant speed of ν and an altitude of *H*. We assume that this altitude is relatively high and that the data transmission duration is reasonably short and denoted by τ. As a result, the UAV is regarded immobile when uploading the UE data.

### 2.2. Wireless Communication Channel Model

For the convenience of designing an emergency wireless communication network, our designed system should utilize a channel in the unlicensed band, i.e., 2.4 GHz. In such a way, this system can be easily integrated with the hardware of modern UEs. Hence, the utilized channel model is expounded at [34], in accordance with the 3rd Generation Partnership Project (3GPP) specification in the technical report presented in [36]. This channel model represents the wireless communication link between the UAV and each of the served UEs into two components, i.e., the line-of-sight (LOS) component and the non-line-of-sight (NLOS) component, according to their corresponding probabilities, and can be calculated by (1).

(1) $$L_{m}=\left\{\begin{array}{cc} 30.9+\left(22.25-0.5\log_{10}H\right)\log_{10}d_{m}+20\log_{10}f, & \mathrm{if}\;\mathrm{LOS}\;\mathrm{link}\\ \max\left(L_{m}^{\mathrm{LOS}},32.4+\left(43.2-7.6\log_{10}H\right)\log_{10}d_{m}+20\log_{10}f\right), & \mathrm{if}\;\mathrm{NLOS}\;\mathrm{link} \end{array}\right.$$

where *H* denotes the UAV flight altitude, *f* is the carrier frequency, and $d_{m}$ is the distance between the UAV and any corresponding UE *m*, which can be calculated as follows:

(2) $$d_{m}=\sqrt{H^{2}+{∥l_{m}-l_{0}∥}^{2}},\;\forall m\in M$$

Since the calculation of path loss due to the NLOS component is a function of the path loss due to the LOS component $L_{m}^{\mathrm{LOS}}$, the term $L_{m}^{\mathrm{LOS}}$ should be calculated prior to estimate the path loss of the NLOS component. The probability of the LOS link is denoted by $P_{m}^{\mathrm{LOS}}$ and given in (3).

(3) $$P_{m}^{\mathrm{LOS}}=\left\{\begin{array}{cc} 1, & \mathrm{if}\sqrt{d_{m}^{2}-H^{2}}\leq d_{0}\\ \frac{d_{0}}{\sqrt{d_{m}^{2}-H^{2}}}+\exp\left\{\left(\frac{-\sqrt{d_{m}^{2}-H^{2}}}{p_{1}}\right)\left(1-\frac{d_{0}}{\sqrt{d_{m}^{2}-H^{2}}}\right)\right\}, & \mathrm{if}\sqrt{d_{m}^{2}-H^{2}}>d_{0} \end{array}\right.$$

(4) $$d_{0}=\max\left(294.05\log_{10}H-432.94,18\right)$$

(5) $$p_{1}=233.98\log_{10}H-0.95$$

Furthermore, the probability of NLOS can be obtained naturally for the probability of LOS as follows:

(6) $$P_{m}^{\mathrm{NLOS}}=1-P_{m}^{\mathrm{LOS}}$$

The channel gain between the UAV and any connected UE can be calculated as follows:

(7) $$g_{m}=P_{m}^{\mathrm{LOS}}{\left(10^{L_{m}^{\mathrm{LOS}}/10}\right)}^{-1}+P_{m}^{\mathrm{NLOS}}{\left(10^{L_{m}^{\mathrm{NLOS}}/10}\right)}^{-1}$$

where $L_{m}^{\mathrm{LOS}}$ and $L_{m}^{\mathrm{NLOS}}$ are the path loss for the LOS and NLOS, respectively, and can be calculated from (1).

### 2.3. Data Transmission Model

For the sake of simplicity, we assumed that the UAV emergency wireless communication network can be established between the UAV and only one UE at any certain time. Hence, there are no simultaneous wireless connections from different UEs to the UAV. The effective radiation angle of the UAV antenna is denoted by φ, so the maximum distance between the UAV and any UE that permits the establishment of a wireless communication link is H/cos(φ). Additionally, it can be observed that the relationship between the channel gain $g_{m}$ in (7) and the distance $d_{m}$ in (2) is an inverse relationship. Therefore, our definition of the effective radiation angle φ is used as a parameter to make sure that this distance is suitable for establishing a wireless communication link. This can be done by evaluating the signal-to-noise ratio (SNR) value for a covered UE. When it reaches a certain threshold that permits the establishment of a wireless communication link, this covered UE can access the UAV to offload its data. Additionally, the value of φ can be chosen to be very narrow to shrink the UAV coverage. In such a way, the simultaneous transmission from different UEs can be easily eliminated. Hence, a UE can be within the UAV coverage if and only if it belongs to the following set:

(8) $$M_{\mathrm{cov}}=\left\{m\in M\;:\;d_{m}\leq H/\cos\left(\varphi \right)\right\}$$

It is assumed that each UE in the post-disaster area has an amount of data equal to Ψ bits. Then, a UE access indicator, denoted by $\alpha_{m}$, is used to show whether the *m*-UE is connected to the UAV or not. This access indicator depends on two factors, i.e., the distance from the UAV, $d_{m}$, and the total uploaded bits from the *m*-UE to the UAV, $\Omega_{m}$. Thus, $\alpha_{m}$ can be expressed as follows:

(9) $$\alpha_{m}\left(t\right)=\left\{\begin{array}{cc} 1, & \mathrm{if}\;m\in M_{\mathrm{cov}},\;\Omega_{m}\left(t\right)<\Psi \\ 0, & \mathrm{otherwise} \end{array}\right.$$

where t∈T,T={1,⋯,T} is the time elapsed while the UAV flies over the post-disaster area. The total uploaded bits from the *m*-UE to the UAV can be calculated as:

(10) $$\Omega_{m}\left(t\right)=\sum_{i=1}^{t}\omega_{m}\left(i\right)$$

where $\omega_{m}$ is the instantaneous uploaded data size at time *t* and can be calculated as follows:

(11) $$\omega_{m}\left(t\right)=R_{m}\left(t\right)\;\tau$$

where $R_{m}$ is the transmission data rate from the *m*-UE to the UAV and can be calculated according to Shannon’s theorem as follows:

(12) $$R_{m}\left(t\right)=\alpha_{m}\left(t\right)\;B\;\log_{2}\left(1+\frac{g_{m}\;P_{m}^{\mathrm{Tx}}}{\sigma_{0}}\right)$$

where *B* is the available wireless channel bandwidth, $P_{m}^{\mathrm{Tx}}$ is the transmission power from *m*-UE, and $\sigma_{0}$ denotes the power of the additive white Gaussian noise (AWGN) at the UAV receiver.

### 2.4. Energy Model

From the perspective of the limited energy capacity, the consumed energy can be classified as follows: (1) the energy consumed by each *m*-UE while it is idle and during the data offloading period; (2) the energy consumed by the UAV while it is flying over the post-disaster area to provide the wireless connectivity for the trapped UEs. Thus, at any time *t*, these two consumed terms of energy can be denoted as follows:

(13) $$e_{m}\left(t\right)=\left\{\begin{array}{cc} \alpha_{m}\left(t\right)\;P_{m}^{\mathrm{Tx}}\;\tau , & \mathrm{if}\;m-\mathrm{UE}\;\mathrm{at}\;\mathrm{Tx}\;\mathrm{mode}\\ \left(1-\alpha_{m}\left(t\right)\right)\;e^{\mathrm{idle}}, & \mathrm{if}\;m-\mathrm{UE}\;\mathrm{at}\;\mathrm{idle}\;\mathrm{mode} \end{array}\right.$$

(14) E(t)=Ξt

where $e^{\mathrm{idle}}$ is the energy consumed by each of *m*-UE during the idle mode, and Ξ is the UAV’s flying power. Of course, there are many factors that control the UAV’s energy consumption, such as the flying speed, acceleration, and mass of the UAV. However, we tried to simplify the notation of the energy consumption to be averaged per unit of time. In such a way, we can study the ability of our proposed solution to handle this dynamic energy consumption over time. Furthermore, the energy consumed by the UAV’s receiver circuit and signal processing are relatively low compared to the energy consumed during flying, so it can be neglected. To expand this research to more detailed power consumption, the work presented in [3] is a straightforward extension, and it will be considered for our future work.

### 2.5. Problem Formulation

The ultimate goal of the post-disaster surveillance system is to improve the rescue success rate of victims and also to reduce casualties. This goal can be achieved by maximizing the data uploaded from the trapped victims in the post-disaster area over the UAV trajectory. At the same time, we must take into account the valuable limited energy of both UEs and the UAV. Mathematically speaking, our optimization problem can be expressed as follows:

(15) $$\begin{array}{cc} \max_{m\in M} & \;\frac{1}{T}\sum_{t=1}^{T}\sum_{m=1}^{M}\omega_{m}\left(t\right) \end{array}$$

(16) $$\begin{array}{cc} s.t.\; & \sum_{t=1}^{T}e_{m}\left(t\right)\leq e_{0},\;\forall m\in M \end{array}$$

(17) $$\begin{array}{cc} \; & \sum_{t=1}^{T}E\left(t\right)\leq E_{0} \end{array}$$

The optimization problem shown in (15) is considered an online optimization problem that aims to maximize the long-term throughput of the whole network by optimizing the UAV’s flight trajectory. Since there is an unlimited number of routes that can be existed by changing the order of how the UAV serves the UEs, our optimization problem is an NP-hard problem. However, by considering energy constraints introduced in Equations (16) and (17), the optimization problem can be viewed as an NP-complete problem. The whole optimization process is done not only in an online manner but also in a decentralized way where there is no information exchange between different network elements. Furthermore, for any conventional programming solvers, all information should be gathered at one centralized entity to solve the optimization problem, which cannot be satisfied while designing an emergency wireless communications network for a post-disaster surveillance system. In such a case, we suggest using a reinforcement-learning-based algorithm to deal with this kind of online optimization problem.

The decision variables can be defined as the accumulated instantaneous throughput $\omega_{m}\left(t\right)$ for all the *M* UEs throughout the UAV’s flight time *T*. The constraint (16) shows that the maximum energy available for each UE is limited by $e_{0}$, and the other constraint (17) limits the energy available for the UAV by $E_{0}$; both are considered the feasibility constraints of the optimization problem. Furthermore, the right-hand sides of constraints (16) and (17) are also long-term cumulative variables related to the UAV flight trajectory. Hence, the whole flight-trajectory process should be taken into account when solving the position of the UAV at any time *t*. Therefore, this optimization problem becomes difficult to figure out using conventional optimization methods. Additionally, sharing information on the remaining battery capacity for every UE in the post-disaster area is quite a changeling, especially when the commercial mobile network has malfunctioned. Therefore, for the sake of simplicity and without loss of generalization, our optimization problem was designed for the worst-case scenario for the available battery capacity for each UE. This value was chosen to be around 10% of modern UE’s average total battery capacity [37]. In the next section, we introduce an MAB-based framework to tackle this issue.

## 3. Dual-Energy-Aware MAB-Based UAV Trajectory Optimization Approach

In this section, we explain the general MAB framework and then illustrate how the proposed dual-energy-aware MAB approach could address our previously described optimization problem.

### 3.1. General MAB Framework

Generally speaking, in any MAB-based framework, a player aims to maximize his long-term reward while playing with a set of arms of the bandit machine, j∈{1,…,J}. This can be performed in a sequential way by selecting an arm at time *t*, i.e., j(t), and observing their corresponding reward, i.e., $r_{j}\left(t\right)$. In the first few moments, the player tries to explore candidate arms as much as possible and observes their corresponding rewards. After that, the player exploits the arm with the highest reward, based on the gathered information from the already explored arms, to maximize the cumulative reward over the episode. This dilemma is quite well-known in the world of the MAB framework and is known as the exploration–exploitation trade-off [15]. The MAB framework can be classified as stochastic or adversarial based on the distribution of the rewards [14,15]. For the stochastic MAB framework, the rewards behind each arm are drawn from independent and identical distribution (i.i.d); however, for the adversarial MAB framework, rewards are selected arbitrarily with no prior distribution. For these two types of MAB frameworks, extensive research has been done to deal with these kinds of problems, resulting in the introduction of different algorithms, such as the ϵ-greedy algorithm [38], the upper confidence bound (UCB) algorithm [39], the Thompson sampling (TS) algorithm [40], and the exponential-weight algorithm for exploration and exploitation (EXP3) [41]. Furthermore, in real-world optimization problems, choosing an arm with a higher reward will have a high cost as well. Thus, cost-effective and budget-constrained MAB algorithms are introduced to deal with these kinds of scenarios [42,43].

### 3.2. The DEA-MAB Approach

To address the online optimization problem with the dynamic energy consumption over time that is given in (15), and which constrained by conditions (16) and (17), an MAB-based framework that is dual-energy-aware called DEA-MAB is proposed. Our DEA-MAB approach is inspired by the cost-subsidized explore-then-commit algorithm proposed in [43], where the chosen arm is accompanied by a certain cost. One of the traditional ways to optimize this reward/cost is to directly deduct the cost from the reward in the control formula. However, this is not usually meaningful in real-world problems, especially when the reward and the cost are defined in different quantities [43], such as the achievable throughput and the energy consumed, as illustrated in our problem formulation. Hence, it is necessary to find a better way to optimize for both the reward and the cost. In other words, the algorithm should avoid incurring an excessive cost for just a marginal increase in the reward. This may be done by building a feasible set of arms which is an estimate of all arms with a mean reward greater than the least tolerable value in each round, based on the upper confidence bound (UCB) and lower confidence bound (LCB) of the reward of each arm. Then, the arm with the lowest cost in this feasible set is selected to be played by it.

Though the cost-subsidized explore-then-commit algorithm is considered a good solution for separating the reward and the cost functions, it still needs some adaptation to tackle our optimization problem that is given in (15). Precisely speaking, our optimization problem considers two different energy costs, so the DEA-MAB algorithm adds a further step for checking the second cost. Thus, some controlling functions were added in the proposed algorithm to precisely address this issue.

Algorithm 1 summarizes how the DEA-MAB algorithm works. The DEA-MAB algorithm’s input attributes are the state spaces of all available M UEs, including their corresponding locations $l_{m}\;\forall \;m\in M$; the total flight time *T*; tuning parameters δ and ϵ; the available energy for each piece of UE $e_{0}$; and the total flight time of the UAV till its battery is completely depleted, *T*. At each time period *t* of the total flight time *T*, the UAV should select one of *M* UEs distributed in the post-disaster area via the DEA-MAB algorithm; then it will fly towards it to offload its data. In the beginning, the algorithm is initialized at t=0 by setting the number of times each *m*-UE is selected, $Q_{m}\left(t\right)$, and their average achievable throughput, ${\bar{\omega }}_{m}\left(t\right)$, to 0. The DEA-MAB algorithm is divided into two phases, i.e., the pure exploration phase and the selection phase. During the exploration phase, the UAV randomly selects a UE to visit as follows:

(18) $$m^{*}\left(t\right)=t\;\mathrm{mod}\;M$$

Then, the corresponding throughput $\omega_{m^{*}}\left(t\right)$ is observed, and the selection number, $Q_{m}\left(t\right)$, and the average throughput, ${\bar{\omega }}_{m}\left(t\right)$, are updated as in the following equations:

(19) $$Q_{m^{*}}\left(t\right)=Q_{m^{*}}\left(t-1\right)+1$$

(20) $${\bar{\omega }}_{m^{*}}\left(t\right)=\frac{1}{Q_{m^{*}}\left(t\right)}\sum_{i=1}^{Q_{m^{*}}\left(t\right)}\omega_{m}\left(i\right)$$

The exploration phase is performed for a time period equal to Mπ, where $\pi ={\left(T/M\right)}^{2/3}$ is as given in [43]. After that, the DEA-MAB algorithm goes for the selection phase during each time t∈[Mπ+1,T], where both the UCB and LCB are calculated as follows:

(21) $$\gamma_{m}^{\mathrm{UCB}}\left(t\right)={\bar{\omega }}_{m}\left(t\right)+\sqrt{2\ln\left(t\right)/Q_{m}\left(t\right)},\;\forall \;m\in M$$

(22) $$\gamma_{m}^{\mathrm{LCB}}\left(t\right)={\bar{\omega }}_{m}\left(t\right)-\sqrt{2\ln\left(t\right)/Q_{m}\left(t\right)},\;\forall \;m\in M$$

Then, the UE index corresponding to the maximum value of the $\gamma_{m}^{\mathrm{LCB}}\left(t\right)$ is calculated as follows:

(23) $$\eta_{t}=\underset{m}{\arg\max}\;\gamma_{m}^{\mathrm{LCB}}\left(t\right)$$

Afterwards, the feasibility region of all UEs having $\gamma_{m}^{\mathrm{UCB}}\left(t\right)\geq \left(1-\delta \right)\gamma_{\eta_{t}}^{\mathrm{LCB}}\left(t\right)$ is enumerated as follows:

(24) $$F\left(t\right)=\left\{m:\gamma_{m}^{\mathrm{UCB}}\left(t\right)\geq \left(1-\delta \right)\gamma_{\eta_{t}}^{\mathrm{LCB}}\left(t\right)\right\}$$

For this set of UEs, F(t), the dissipated energy for each of the *m*-UE contained in this F(t) list is obtained. Then, a control set, C(t), is constructed out of all UEs in F(t). A check is performed for the UEs’ energy consumption; then priority is given to all UEs in the F(t) list in case they exceed their energy consumption with a value of 1−ε of the total available energy $e_{0}$. Otherwise, C(t) is set to be equal to F(t). This can be illustrated as follows:

(25) $$C\left(t\right)=\left\{\begin{array}{cc} m:\sum_{i=1}^{t}e_{m}\left(i\right)\geq \left(1-\varepsilon \right)e_{0}, & \sum_{i=1}^{t}e_{m}\left(i\right)\geq \left(1-\varepsilon \right)e_{0}\\ F(t), & \mathrm{otherwise} \end{array}\right.$$

Out of this list, C(t), the UE corresponding to the minimum UAV energy cost, E(T), is selected as a next-served UE for data offloading in the UAV flight trajectory as follows:

(26) $$m^{*}\left(t\right)=\underset{m\in C(t)}{\arg\min}\;E\left(t\right)$$

Afterwards, values of the selection number, $Q_{m}\left(t\right)$, and the average throughput, ${\bar{\omega }}_{m^{*}}\left(t\right)$, are updated for the selected UE, $m^{*}\left(t\right)$, as given in Algorithm 1. Since the UAV should accomplish the whole data offloading task and ensure flying back to its base before the battery is used up, the UAV should confirm that there is enough remaining battery energy for returning. Otherwise, the UAV could be lost or damaged if it cannot arrive at its base before the battery becomes empty. Therefore, a checking step is provided to confirm this critical condition at each time before deciding to choose the next UE to be served. In this way, the DEA-MAB algorithm can optimize the UAV’s flight trajectory considering limited energy of both the UAV and the UEs.

**Algorithm 1:** The proposed algorithm: DEA-MAB.

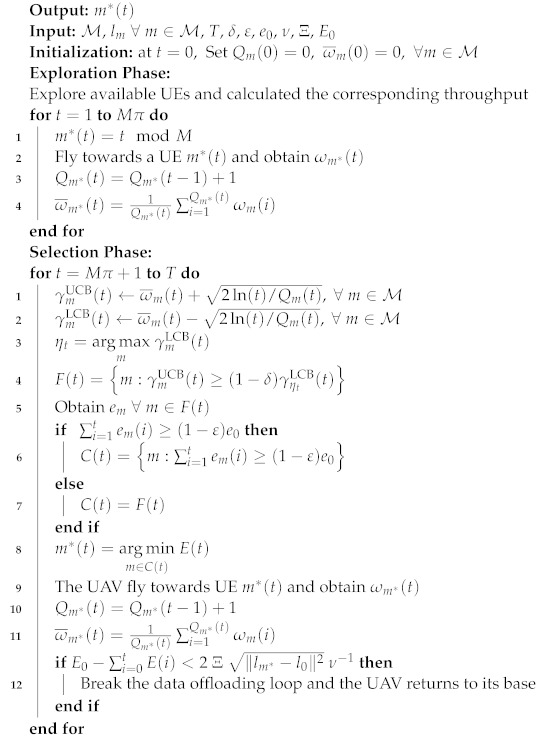



### 3.3. Complexity Analysis of the Proposed Approach

In the previous section, the task of the UAV finding the best trajectory in the post-disaster area is spotlighted. This is accomplished by finding the optimal policy to choose the next UE to be served through the learning process in Algorithm 1. In the beginning, the uplink throughput that can be achieved while the UAV connects to this UE is examined. Then, a higher priority is given to UEs whose batteries are nearly depleted. The consumed energy during UAV flying is also minimized. Moreover, it is assumed that the action space is deterministic; i.e., all actions are well-known to the UAV. Hence, the fundamental source of the computational complexity of the DEA-MAB algorithm comes from calculating both the UCB and the LCB. Then, other parameters are updated according to this selection. It should be mentioned that these parameters have the same computational complexity order as UCB or LCB. Hence, the overall computational complexity order of our proposed algorithm is a polynomial of M+1, and can be expressed as O(M+1) [43].

## 4. Simulation Results

In this section, the performance of the DEA-MAB algorithm is evaluated. In the simulation, it was assumed that the UAV will provide wireless connectivity for a previously allocated area where there are *M* trapped UEs which are randomly distributed. However, for a large post-disaster area, more than one UAV can be deployed to support the data offloading while considering the coordination between UAVs to facilitate rescue operations. This larger system is left for future work.

Table 1 shows the simulation parameters used in verifying our proposed algorithm. In order to investigate the effectiveness of our proposed framework, two trajectory optimization methods were used as benchmarks for the sake of comparison. These two methods can be described as follows:

1. The post-disaster area spiral scanning (PASS) method: This method is designed to scan the whole area using the spiral path where the UAV starts to fly from the center of the post-disaster area. With respect to the UAV antenna’s radiation angle, a projected circle is created on the ground. This circle scans the whole post-disaster area from the center to the borders.
2. Shortest flight path (SFP) method: In this method, the UAV starts to fly from the center of the post-disaster area and then selects the UE with the shortest path. Then, the UAV flies toward this UE and hovers above it to offload its data. After that, the UAV searches for the next close UE and flies toward it. This operation is performed till the last UE.

In the following, the performance of the proposed framework is evaluated by comparing it with benchmark algorithms during the varying of both the number of trapped UEs in the post-disaster area and the UAV’s battery capacity. For the sake of accuracy, and due to the randomness in UEs’ distributions, all simulations were performed for a long enough time, i.e, $10^{4}$ iterations. The average value of each case is provided for a better estimation of the result.

**Figure 2:**
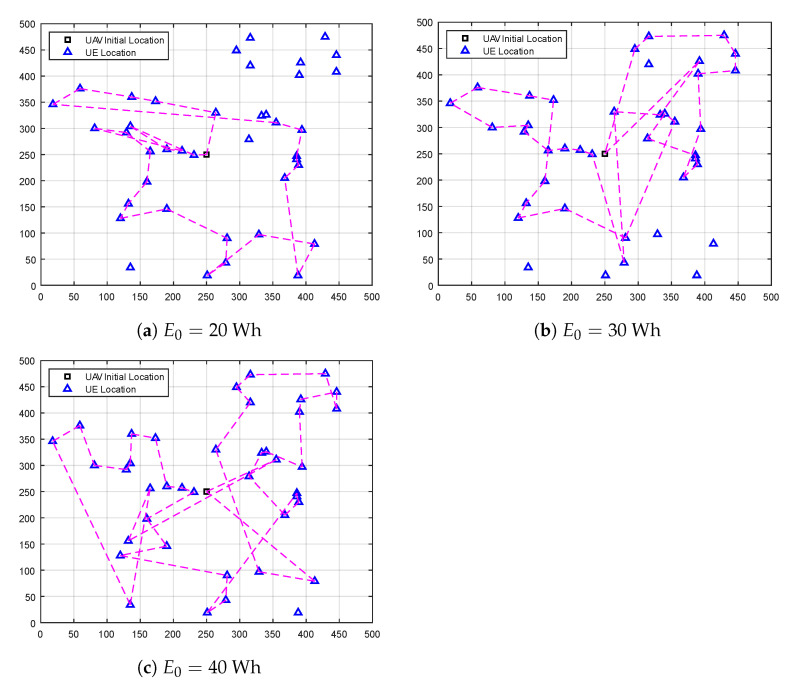
A sample of the UAV flight trajectory using the DEA-MAB algorithm.

Figure 2 shows a sample of the UAV’s flight trajectory in the post-disaster area. To visualize how our DEA-MAB algorithm could optimize the UAV’s flight trajectory considering its available battery power, three different values were used, i.e., $E_{0}=20,30,40$ Wh, while keeping the number of UEs equal to 40. Obviously, increasing available UAV battery power increases the chance of serving more UEs in the post-disaster area.

**Figure 3:**
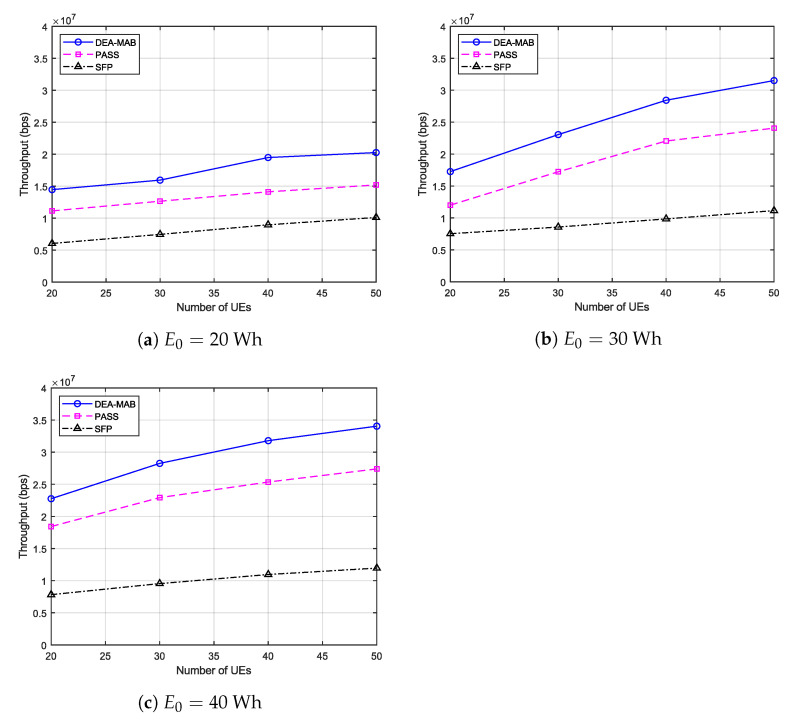
The DEA-MAB algorithm’s throughput versus the number of users.

Figure 3 gives the long-term throughput for the data uploaded from UEs in the emergency wireless communication network. It is clearly visible that regardless of the value of the UAV’s battery capacity or the algorithm used, as the number of UEs trapped in the post-disaster area increases, the uplink data throughput increases as well. Nevertheless, this upward trend gradually decreases, and all curves would saturate at a certain number of UEs. This is because the maximum capacity of a communication system with a fixed bandwidth is fixed. Hence, while the number of UEs increases, the accumulated uplink throughput of the emergency wireless network continues to approach this maximum capacity. When comparing the throughput performance of the DEA-MAB algorithm with other benchmark methods at various values of UAV battery capacity, it is clear that our proposed algorithm can achieve more uplink throughput than the PASS method, and much higher than the SFP method. For example, when ($E_{0}=20$ Wh, M=30), ($E_{0}=30$ Wh, M=40), and ($E_{0}=40$ Wh, M=50), the DEA-MAB algorithm achieved higher throughput performance by 26%, 28%, and 24% compared to the PASS method, and high performance by 113%, 188%, and 184% than the SFP method, respectively.

**Figure 4:**
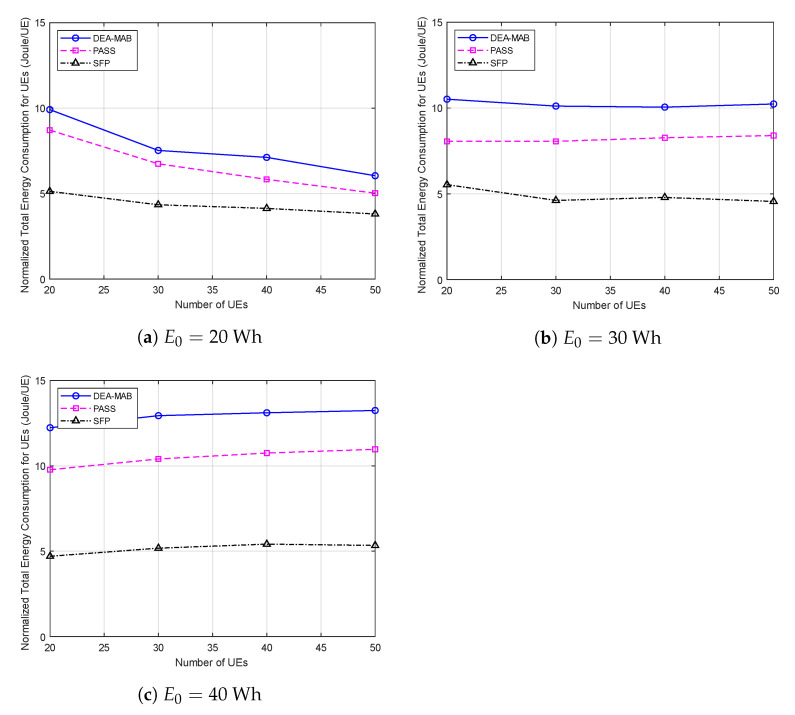
Normalized total energy consumption versus the number of users.

In Figure 4, the normalized total energy consumption of all UEs trapped in the post-disaster area is compared among the three methods. It can be seen clearly that regardless of the used method, as the number of UEs increases, the total normalized energy consumption of UEs increases as well. Furthermore, for the same method with a certain number of UEs, the higher the UAV’s battery capacity, the more energy consumed per UE. This can be justified, as when the UAV has a higher battery capacity, it can have a higher chance to offload data from a larger number of UEs before its battery becomes depleted. Additionally, since $P_{m}^{\mathrm{Tx}}\tau >>e^{\mathrm{idle}}$, more UEs tend to consume energy in the data-offloading process rather than just staying in idle mode. When comparing the normalized energy consumption performance of the DEA-MAB algorithm with other benchmark methods at the same values of UAV battery capacity, it can be shown that the DEA-MAB algorithm always has higher energy consumption than the PASS method, and much higher than the SFP method. This can be explained by the overall system throughput being increased at the cost of more energy consumption by the UEs. For the sake of comparison, let us observe the same points at ($E_{0}=20$ Wh, M=30), ($E_{0}=30$ Wh, M=40), and ($E_{0}=40$ Wh, M=50): the total energy consumption of all UEs using the DEA-MAB algorithm was increased by 11%, 24%, and 23% compared to the PASS method, and by 73%, 109%, and 169% compared to the SFP method.

As observed from the analysis of results in Figure 3 and Figure 4, it can be concluded that the DEA-MAB algorithm can achieve a considerable increase in the uplink throughput of UEs with a reasonable increase in the UEs energy consumption. Hence, for a better understanding of the advantages of using the DEA-MAB algorithm, the UEs’ energy efficiency (μ) is compared using our proposed algorithm against benchmark methods. μ can be defined as the ratio of the long-term UEs uplink throughput over the total UEs energy consumption in bit/Joule as follows:

(27) $$\mu =\frac{\sum_{t=1}^{T}\sum_{m=1}^{M}\omega_{m}\left(t\right)}{\sum_{t=1}^{T}\sum_{m=1}^{M}e_{m}\left(t\right)}$$

**Figure 5:**
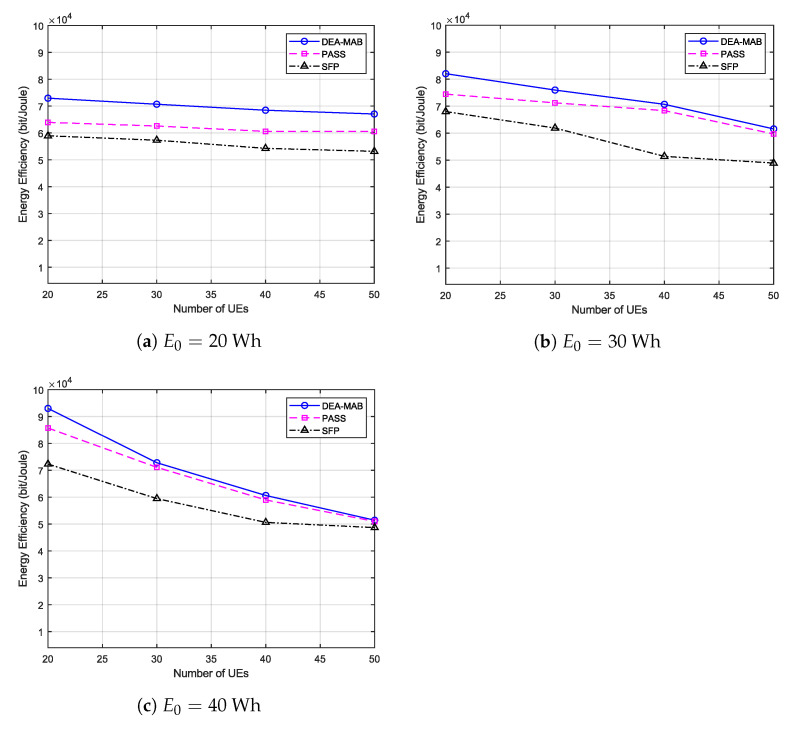
UEs’ energy efficiency versus the number of users.

In the energy efficiency performance shown in Figure 5, it is observed clearly that whatever the UAV’s battery capacity or the number of UEs trapped in the post-disaster area, the DEA-MAB algorithm can surpass benchmark methods in terms of energy efficiency, which, of course, means enhancing the overall performance of the emergency wireless communication network. It should be mentioned that, when increasing the UAV’s battery capacity to 40 Wh, as in Figure 5c, the PASS method achieved a performance that is very close to that of the DEA-MAB algorithm. This can be justified, as the UAV’s battery at this point becomes quite enough to accomplish the spiral scanning for a major part of the post-disaster area.

## 5. Conclusions

In this paper, the trajectory optimization for a UAV-assisted emergency wireless communication network was investigated. The UAV is deployed as a temporary BS to provide wireless connectivity from the sky for trapped UEs in a post-disaster area where all BSs are damaged or have malfunctioned due to a natural disaster. The UAV’s target is to optimize its flying trajectory to maximize the long-term uplink throughput from UEs. However, due to the malfunctioning of the power supplies in the disaster area as well, this optimization problem is performed with limited battery capacity of not only the UAV but also UEs in the post-disaster area. We proposed an MAB-based algorithm constrained with these two energy limitations to address this optimization problem. The proposed algorithm can solve the trajectory optimization problem with respect to this dynamic energy consumption over time. Simulation results showed that our algorithm outperforms benchmark methods in terms of long-term uplink throughput and energy efficiency. Furthermore, it could increase the energy consumption of the UEs during the data offloading process, which reflects success in maximizing the UEs served in a post-disaster area and accomplishing the task of information collection in the post-disaster area. A straightforward extension could be to expand the simulation area to be served with more than one UAV. In such a case, each UAV would have to develop a strategy to not only maximize the objective function but also to avoid collisions with other UAVs. One of these strategies would be to keep a certain operating distance between each pair of UAVs. This distance could be designed using optical sensors attached to the UAV to recognize the surrounding UAVs, or by detecting a low-power beacon signal transmitted from each operating UAV. A detailed system design was kept for our future work. Additionally, for a more realistic scenario, UEs might be considered as moving objects, and the UAV should consider an accurate methodology for estimating the location of each UE that should be served.

## Figures and Tables

**Table 1:** Simulation parameters.

Parameter	Value
Simulation area	500 m × 500 m
Number of UEs in the simulation area (*M*)	20,30,40,50
UAV flight speed (ν)	20 km/h
UAV flight altitude (*H*)	100 m
UAV antenna radiation angle (φ)	π/8 rad
Carrier frequency (*f*)	2.4 GHz
Channel bandwidth (*B*)	10 MHz
Data transmission duration (τ)	1 s
UE Transmission power ($P_{m}^{\mathrm{Tx}}$)	23 dBm
AWGN spectral density ($\sigma_{0}$)	−130 dBm/Hz
UAV battery capacity ($E_{0}$)	20,30,40 Wh
UAV flying power (Ξ)	120 W
UE battery capacity ($e_{0}$)	1 Wh
UE energy dissipation in idle mode ($e^{\mathrm{idle}}$)	0.01 J
Data rate feasibility region factor (δ)	0.6
Critical power feasibility region factor (ε)	0.5

## Data Availability

Not applicable.

## Abbreviations

The following abbreviations are used in this manuscript:

BS	Base station
UAV	Unmanned aerial vehicle
ML	Machine learning
RL	Reinforcement learning
MAB	Multi-armed bandit
DPG	Deterministic policy gradient
MDP	Markov decision process
CRN	Cognitive radio network
UCB	Upper confidence bound
TS	Thompson sampling
RIS	Re-configurable intelligent surface
SUTOA	state-action-reward-state-action based UAV-trajectory optimization algorithm
QUTOA	Q-learning based UAV-trajectory optimization algorithm
UE	User equipment
GPS	Global positioning system
3GPP	3rd generation partnership project
LOS	Line-of-sight
NLOS	Non-line-of-sight
SNR	Signal-to-noise ratio
AWGN	Additive white Gaussian noise
EXP3	The exponential-weight algorithm for exploration and exploitation
LCB	Lower confidence bound
DEA	Dual-energy aware
PASS	Post-disaster area spiral scanning
SFP	Shortest flight path
PoI	Points of interest
MILP	Mixed Integer Linear Programming
